# Investigation on Design Theory and Performance Analysis of Vacuum Capacitive Micromachined Ultrasonic Transducer

**DOI:** 10.3390/mi12091127

**Published:** 2021-09-19

**Authors:** Xiao Huang, Hongliang Wang, Lijun Yu

**Affiliations:** 1 National Key Laboratory for Electronic Measurement Technology, North University of China, Taiyuan 030051, China; huangxiao.nuc.edu@outlook.com (X.H.); weilijun1997@foxmail.com (L.Y.); 2 Key Laboratory of Instrumentation Science & Dynamic Measurement, North University of China, Taiyuan 030051, China

**Keywords:** vacuum, capacitive micromachined ultrasonic transducer (CMUT), finite element simulation, structural design, performance influence, performance optimization

## Abstract

The capacitive micromachined ultrasonic transducer (CMUT), as a new acoustic-electric conversion element, has a promising application prospect. In this paper, the structure of the vacuum capacitive micromachined ultrasonic transducer is presented, and its performance-influencing factors are investigated. Firstly, the influencing factors of the performance parameters of the vacuum CMUT are analyzed theoretically based on the circular plate model and flat plate capacitance model, and the design principles of the structural parameters of the CMUT cell are proposed. Then, the finite element simulation software COMSOL Multiphysics is used to construct CMUT cell models with different membrane materials, membrane shapes, membrane radius thicknesses, and cavity heights for simulation verification. The results show that both the membrane parameters and the cavity heights affect the performance parameters of the Vacuum CMUT. In order to improve the efficiency of the CMUT, materials with low bending stiffness should be selected, and the filling factor of the membrane should be increased. In order to achieve high-transmission sound pressure, a smaller radius thickness and a larger cavity height should be selected. To achieve high reception sensitivity, a larger membrane radius thickness and a smaller cavity height should be selected. In order to obtain high fractional bandwidth, a larger membrane radius thickness should be selected. The results of this paper provide a basis for the design of Vacuum CMUT cell structure.

## 1. Introduction

With the development of micro-electro-mechanical systems (MEMS) technology, the capacitive ultrasonic micromachined transducer (CMUT) research has become an important direction of ultrasonic transducer research. Compared with the piezoelectric ultrasonic transducer (PMUT), the CMUT presents the advantages of wide bandwidth, high resolution, great acoustic impedance matching, easy integration with front-end circuits, and easy mass production, which has gradually become a substitute for PMUT [1,2,3,4,5,6,7,8,9].

The traditional CMUT will produce losses and affect its efficiency in the working process. Hayrettin Köymen and Muhammed N. Senlik et al. present a lumped element parametric model for the clamped circular membrane of a CMUT. The model incorporates an electrical port and two sets of acoustic ports through which the CMUT couples to the medium. The modeling approach is based on matching a lumped element model and the mechanical impedance of the CMUT membrane at the resonance frequencies in vacuum. Very good agreement between finite element simulation results and model impedance is obtained [10]. In order to improve the working efficiency of the CMUT, the vacuum CMUT is proposed in this paper, the cavity of which is vacuum sealed. The CMUT generally works in an air or liquid environment, with different sources of energy loss in different environments. When the CMUT works in the air, its energy loss comes from viscous damping, acoustic radiation, heat conduction, compressed gas damping, membrane tension, etc., among which the compressed gas damping of the cavity is the main influencing factor, and a Vacuum CMUT can reduce such loss to the greatest extent. When the working medium of the CMUT is liquid, the loss resulted from hydrolyzation caused by the strong electric field in the cavity is the main source of loss. Vacuum sealing of the cavity can reduce this loss. In conclusion, vacuum CMUT can reduce the loss of the CMUT and thus improve the work efficiency. Its design and investigation are of great significance. Vacuum CMUT can be widely used in many fields such as intravascular ultrasound detection [11], intravascular photoacoustic imaging [12], tissue harmonic imaging [13], high-intensity focused ultrasound therapy [14], and liquid flow velocity measurement [15].

In the initial stage of CMUT design, it is necessary to model and simulate to verify the correctness of the design parameters. Satir and Zahorian et al. developed a large-signal, transient model to predict the output characteristics of a CMUT array operated in the non-collapse mode. The model is based on the separation of the nonlinear electrostatic voltage-to-force relation and the linear acoustic array response [16]. Using analytical calculations and FEA as the tuning tool, Maadi and Zemp developed a precise large signal equivalent circuit model of square CMUT dynamics, and it showed excellent agreement with finite-element modeling (FEM) results [17]. Lohfink and Eccardt describe in detail a new method, which derives a 1D model for CMUT arrays from finite-element methods (FEM) simulations. As a main advantage, the nonlinear behavior of the CMUT can be investigated much easier and faster compared to FEM simulations, e.g., for a design of the maximum applicable voltage depending on the input signal. The commonly simulation models include the equivalent circuit model [18] and finite element simulation model [19]. The equivalent circuit model expresses the main performance parameters of CMUT using components in the circuit system according to the energy conversion relation of the CMUT. The modeling process of this simulation method is complicated, and ideal approximate conditions are used in the modeling process, so the calculation results are not accurate enough to reflect the performance of the CMUT. Finite element simulation is to construct the geometry, set the boundary conditions and physical field conditions in the finite element simulation software, and use the software’s multi-physics interaction to obtain the performance parameters of the CMUT. This method is simple in modeling and accurate in calculation. It can also be used in irregular shapes and is widely used in the research of the properties of the CMUT cell.

As an acoustic–electric conversion element, the CMUT’s working efficiency is characterized by its performance parameters such as transmission sound pressure, reception sensitivity, and fractional bandwidth [12]. In the design process, it is necessary to optimize the structural parameters that affect these performances to improve the efficiency of the CMUT. This paper mainly analyzes the influencing factors of various performance parameters of the Vacuum CMUT, puts forward the design principles of each structural parameter, and uses the finite element simulation software COMSOL Multiphysics to analyze the influence of the materials, the shape, the radius thickness of the membrane, and the height of the cavity on the performances of vacuum CMUT. The investigation provides a basis for the design and manufacture of Vacuum CMUT.

## 2. Theoretical Analysis of Vacuum CMUT

### 2.1. Vacuum CMUT Cell Structure

**Figure 1:**
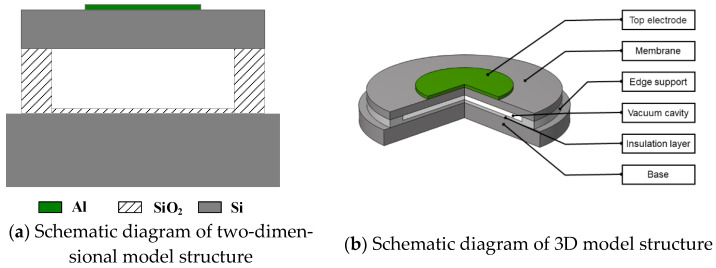
Vacuum CMUT cell structure diagram.

The Vacuum CMUT is composed of several CMUT cells, and the performance of CMUT cells directly determines the performance of the CMUT. This paper mainly analyzes the influence of structural parameters on the performance of the CMUT from the perspective of cells. In general, the Vacuum CMUT cell is generally composed of an electrode on the metal, membrane, edge support, vacuum cavity, insulation layer, and base [20]. The membrane is usually designed into regular shapes such as circle, squares, hexagons, etc. Figure 1 shows the structure diagram of a cell using a circular membrane.

### 2.2. Vacuum CMUT Design Principle

#### 2.2.1. Frequency

Frequency is the primary consideration in the design of the Vacuum CMUT. When the frequency of the applied excitation signal is equal to the frequency of CMUT, the membrane begins to resonate, and a larger deflection is achieved. At this point, the CMUT acquires relatively large sound pressure and reception sensitivity, and the conversion efficiency is optimized.

The frequency of the CMUT is related to the radius and thickness of the membrane. The circular plate model is usually used to study the relationship between the frequency and the radius with the thickness of the membrane [21]. In general, the membrane thickness is very small, and it can be assumed that the membrane stress is uniformly distributed along the thickness direction in the analysis. The vibration equation of the membrane is as follows:

(1) $$\frac{EK^{2}}{\rho (1-\sigma^{2})}\nabla^{4}\eta +\frac{\partial^{2}\eta }{\partial t^{2}}=0,\nabla^{4}={\left(\frac{\partial^{2}}{\partial x^{2}}+\frac{\partial^{2}}{\partial y^{2}}\right)}^{2}=(\frac{\partial^{4}}{\partial x^{4}}+2\frac{\partial^{2}}{\partial x^{2}}\frac{\partial^{2}}{\partial y^{2}}+\frac{\partial^{4}}{\partial y^{4}})$$

where *E* is the Young’s modulus of the membrane material, *σ* is the Poisson’s ratio, *η* is the vibration displacement, and *K* is the cross-section radius of the membrane.

The variable *η* is separated to:

(2) $$\eta (t,x,y)=\eta_{a}(x,y)e^{i\omega t}.$$

Substituting the above equation into Equation (1) to obtain the differential equation:

(3) $$(\nabla^{4}-k^{4})\eta_{a}=(\nabla^{2}+k^{2})(\nabla^{2}-k^{2})\eta_{a}=0$$

where:

(4) $$k^{4}=\frac{\omega^{2}\rho (1-\sigma^{2})}{K^{2}E}.$$

The differential equation can be solved as follows:

(5) $$\eta_{a}=AJ_{0}(kr)+BI_{0}(kr)$$

among them, *J*_0_(*kr*) and *I*_0_(*kr*) are the zero-order cylindrical Bessel function and zero-order imaginary variable cylindrical Bessel function, and *A* and *B* are constants.

The boundary of the membrane is the peripheral fixed support, and the displacement and velocity are both 0, namely:

(6) $$\left\{ \begin{array}{l} \eta_{a}|{}_{r=a}=0\\ \frac{\partial \eta_{a}}{\partial r}|{}_{r=a}=0 \end{array}\right..$$

Substituting this boundary condition into Equation (2):

(7) $$\left\{ \begin{array}{l} AJ_{0}(ka)+BI_{0}(ka)=0\\ -AJ_{1}(ka)+BI_{1}(ka)=0 \end{array}\right..$$

*J*_1_(*ka*) and *I*_1_(*ka*) are the first-order cylindrical Bessel function and first-order imaginary variable cylindrical Bessel function, since *A* and *B* cannot be zero at the same time, therefore:

(8) $$\left|\begin{array}{l} J_{0}(ka)\;I_{0}(ka)\\ -J_{1}(ka)\;I_{1}(ka) \end{array}\right|=J_{0}(ka)I_{1}(ka)+J_{1}(ka)I_{0}(ka)=0.$$

Finally, the frequency of the circular membrane Vacuum CMUT can be obtained as follows:

(9) $$f_{n}=\frac{\mu_{n}^{2}h}{4\pi a^{2}}\sqrt{\frac{E}{3\rho (1-\sigma^{2})}}$$

where *a* is the membrane radius, *h* is the membrane thickness, *ρ* is the membrane density, *E* is the membrane Young’s modulus, *σ* is the membrane Poisson’s ratio, and *μ_n_* is the solution of the *n*-th order cylindrical Bessel function, which is often taken as *μ*_1_ = 3.2, *μ*_2_ = 6.3, and *μ*_3_ = 9.44. The first-order frequency of the Vacuum CMUT can be obtained as follows:

(10) $$f_{1}=\frac{\mu_{1}^{2}h}{4\pi a^{2}}\sqrt{\frac{E}{3\rho (1-\sigma^{2})}}=\frac{0.467h}{a^{2}}\sqrt{\frac{E}{\rho (1-\sigma^{2})}}.$$

It can be seen from the above equation that the frequency of the Vacuum CMUT is determined by the membrane radius and thickness. When the membrane material is determined, the larger the radius is, the smaller the thickness is, and the larger the frequency is. When Vacuum CMUT cell is designed, a specific frequency can be achieved by selecting different combinations of membrane radius and thickness.

#### 2.2.2. Membrane Deflection

The membrane deflection is an important parameter in Vacuum CMUT operation, which determines the capacitance of CMUT. When the membrane is not deformed, the CMUT cell structure is equivalent to a parallel plate capacitor, and the static capacitance of the Vacuum CMUT can be calculated by the relative area of the electrodes and the height of the cavity. When the membrane is deformed by external excitation, the CMUT cell structure can be regarded as consisting of countless small capacitors. The distance between the plates of each small capacitor can be calculated through the deflection of the membrane, thus obtaining the dynamic capacitance of Vacuum CMUT [22]. By increasing the membrane deflection, the capacitance variation of CMUT can be increased, so as to improve the efficiency of Vacuum CMUT.

According to the plate and shell theory, the differential equation of circular membrane with radius *a* subjected to uniform load *q* can be obtained:

(11) $$D\left(\frac{d}{dr^{2}}+\frac{1}{r}\frac{d}{dr}+\frac{1}{r^{2}}\frac{d^{2}}{d\theta }\right)\left(\frac{d^{2}x}{dr^{2}}+\frac{1}{r}\frac{dx}{dr}+\frac{1}{r^{2}}\frac{d^{2}x}{d\theta^{2}}\right)=q$$

where the center of the circular membrane is the origin of coordinates, r is the distance from the origin, x is the deflection of the membrane, and *D* is the bending stiffness of the membrane:

(12) $$D=\frac{Eh^{3}}{12(1-\sigma^{2})}.$$

*E* is the membrane Young’s modulus, and σ is the membrane Poisson’s ratio.

Since the membrane is axisymmetrical and the load is uniform, the deflection equation is independent of the angle θ. Equation (11) can be simplified as:

(13) $$D\left(\frac{d}{dr^{2}}+\frac{1}{r}\frac{d}{dr}\right)\left(\frac{d^{2}x}{dr^{2}}+\frac{1}{r}\frac{dx}{dr}\right)=q.$$

The general solution of the differential equation can be obtained by solving the above equation:

(14) $$x=\frac{qr^{4}}{64D}+\frac{C_{1}r^{2}}{4}+C_{2}\log\frac{r}{a}+C_{3}.$$

The membrane is edge fixed, and its boundary conditions are as follows:

(15) $$\left(\frac{qr^{4}}{64D}+\frac{C_{1}r^{2}}{4}+C_{2}\log\frac{r}{a}+C_{3}\right)|{}_{r=a}=0$$

(16) $$\left(\frac{\partial x}{\partial r}\right)|{}_{r=a}=0,\left(\frac{\partial x}{\partial r}\right)|{}_{r=0}=0.$$

Substituting the boundary conditions into Equation (14):

(17) $$C_{1}=-\frac{qa^{2}}{8D},C_{2}=0,C_{3}=\frac{qa^{4}}{64D}.$$

Thus, the deflection expression at each point of the membrane can be obtained:

(18) $$x(r)=\frac{q}{64D}{\left(a^{2}-r^{2}\right)}^{2}.$$

It can be seen from the above equation that the deflection of the membrane is related to the load and the bending stiffness of the membrane. When the load is constant, the smaller the bending stiffness of the membrane is, the greater the deflection is. Therefore, materials with low bending stiffness should be selected in the design of the Vacuum CMUT cell.

#### 2.2.3. Transmission Sound Pressure

The transmission sound pressure is an important index to measure the transmission performance of the Vacuum CMUT. When the CMUT is used as a transmitter, the transmission sound pressure should be increased as much as possible.

**Figure 2:**
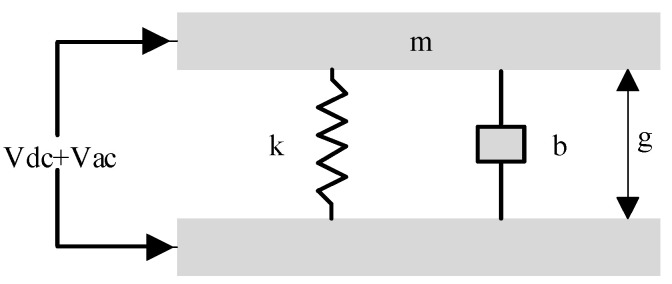
Parallel plate capacitance model (transmitted).

The parallel plate capacitance model is usually used to investigate the relationship between the transmission sound pressure and the structure of the Vacuum CMUT cell [23], as shown in Figure 2, where *m* and *k* are the equivalent mass and equivalent spring coefficient of the membrane, *b* is the damping coefficient, *g* is the cavity height, and *Vdc* and *Vac* are the voltage signals applied to the Vacuum CMUT cell. When the CMUT is in the transmitting state, a DC bias voltage *Vdc* and an AC excitation voltage *Vac* are applied between the upper and lower plates. Under the action of the AC signal, the membrane vibrates up and down, pushing the surrounding medium to do work, which produces ultrasonic waves.

According to Newton’s second law, the force analysis of the membrane can be obtained as follows:

(19) $$mx^{\prime \prime }+bx^{\prime }+kx=Fe$$

where *Fe* is electrostatic force:

(20) $$Fe=\frac{\varepsilon_{0}A{\left(V_{dc}+V_{ac}\right)}^{2}}{2{\left(g-x\right)}^{2}}=\frac{\varepsilon_{0}A}{2{\left(g-x\right)}^{2}}(V_{dc}^{2}+V_{ac}^{2}+2V_{dc}V_{ac}).$$

Here, the higher-order harmonics can be ignored. The above equation is simplified and substituted into Equation (19), which can be obtained as follows:

(21) $$mx^{\prime \prime }+bx^{\prime }+kx=\frac{\varepsilon_{0}AV_{dc}V_{ac}}{{\left(g-x\right)}^{2}}$$

where electric field intensity *E*_0_ and capacitance *C*_0_ are respectively expressed as:

(22) $$E_{0}=\frac{V_{dc}V_{ac}}{g-x},C_{0}=\frac{\varepsilon_{0}A}{g-x},n=E_{0}C_{0}.$$

Therefore, the transmission sound pressure of the CMUT can be expressed as:

(23) $$p_{max}=\frac{n}{A}=\frac{\varepsilon_{0}V_{dc}V_{ac}}{{\left(g-x\right)}^{2}}.$$

It can be seen that Vacuum CMUT transmission sound pressure is mainly related to the cavity height of cell. In order to obtain a higher transmission sound pressure, the cavity height should be increased as much as possible.

#### 2.2.4. Reception Sensitivity

The reception sensitivity is usually used to characterize and measure the reception performance of Vacuum CMUT. When CMUT is used as a receiver, the reception sensitivity should be increased as much as possible.

**Figure 3:**
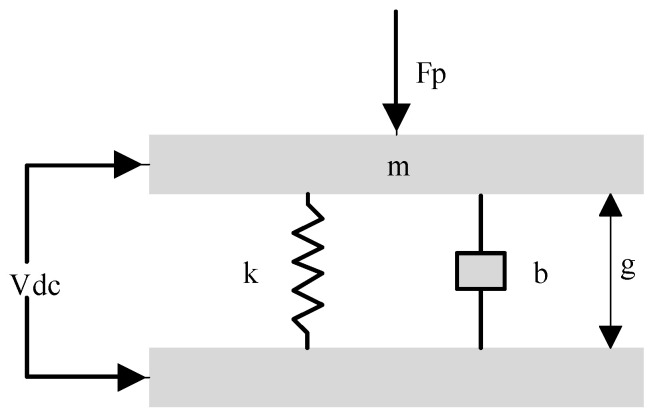
Parallel plate capacitance model (received).

In the receiving state, the parallel plate capacitance model shown in Figure 3 is adopted to investigate the reception sensitivity of Vacuum CMUT [24]. DC bias voltage *Vdc* is applied between the upper and lower plates of the CMUT, while the CMUT is excited by external ultrasonic wave. Under the action of ultrasonic waves, the membrane vibrates up and down, and the distance between the upper and lower plates of CMUT changes, causing a change of capacitance.

The force of the membrane is shown in Equation (24):

(24) $$mx^{\prime \prime }+bx^{\prime }+kx=Fe+Fp$$

where *Fe* is the electrostatic force generated by the DC bias voltage $V_{dc}$, and its value is:

(25) $$Fe=\frac{\varepsilon_{0}AV_{dc}{}^{2}}{2{\left(g-x\right)}^{2}}.$$

Substituting Equation (24) to sort out:

(26) $$Fp=mx^{\prime \prime }+bx^{\prime }+kx-\frac{\varepsilon_{0}AV_{dc}{}^{2}}{2{\left(g-x\right)}^{2}}.$$

The variation of CMUT capacitance can be expressed as:

(27) $$\Delta C=\frac{\varepsilon_{0}A}{g-x}-\frac{\varepsilon_{0}A}{g}=\frac{\varepsilon_{0}Ax}{(g-x)g}.$$

The reception sensitivity *S* of CMUT can be expressed as the ratio of capacitance variation to the receiving sound pressure, namely:

(28) $$S=\frac{\Delta C}{P}=\frac{\varepsilon_{0}Ax}{P(g-x)g}.$$

It can be concluded from the above equation that the reception sensitivity of the Vacuum CMUT is related to the cavity height and the membrane area. The smaller the cavity height and the larger the membrane area, the greater the receiving sensitivity of the Vacuum CMUT.

#### 2.2.5. Fractional Bandwidth

Fractional bandwidth is the ratio of the corresponding frequency range to the center frequency when the amplitude on the amplitude-frequency characteristic curve is attenuated by 3 dB, and it is one of the important performance parameters of the Vacuum CMUT. Different applications require different bandwidth. Ultrasonic imaging requires a wide bandwidth of the CMUT, while gas sensing requires a smaller bandwidth.

The influencing factor of bandwidth is mechanical impedance and acoustic impedance [25]. When the CMUT is operating in the air, the bandwidth is mainly determined by the mechanical impedance of the vibration membrane. Mechanical impedance is usually defined as the ratio of the load on the membrane surface to the vibration velocity, as shown in the following equation:

(29) $$Z_{m}=\frac{p}{\bar{v}}=j\omega \rho h\left[\frac{ak_{1}k_{2}(k_{1}I_{0}(k_{2}a)J_{1}(k_{1}a)+k_{2}I_{1}(k_{2}a)J_{0}(k_{1}a))}{ak_{1}k_{2}(k_{1}I_{0}(k_{2}a)J_{1}(k_{1}a)+k_{2}I_{1}(k_{2}a)J_{0}(k_{1}a))-2(k_{1}^{2}+k_{2}^{2})J_{1}(k_{1}a)I_{1}(k_{2}a)}\right].$$

*k*_1_ and *k*_2_ are respectively given by:

(30) $$k_{1}=\sqrt{\frac{\sqrt{d^{2}+4c\omega^{2}}-d}{2c}},k_{2}=j\sqrt{\frac{\sqrt{d^{2}+4c\omega^{2}}+d}{2c}}$$

where:

(31) $$c=\frac{(E+T)h^{2}}{12\rho (1-\delta^{2})},d=\frac{T}{\rho }.$$

The smaller the mechanical impedance, the wider the Vacuum CMUT bandwidth and vice versa. In the design process of the Vacuum CMUT cell, the mechanical impedance can be adjusted by changing the radius and thickness of the membrane to achieve the appropriate bandwidth.

## 3. Finite Element Analysis of Vacuum CMUT

The finite element simulation method uses mathematical approximation for modeling and analysis. This method uses simple models to replace more complex practical problems, and the solution process is relatively simple. In the solution process, the solution domain is divided into many small continuous units, and the solution is performed on each small unit; then, the solutions of these small units are analyzed and calculated, and finally, the solution of the whole solution domain is obtained [26].

The simulation software used in this paper is COMSOL Multiphysics 5.4 (COMSOL Inc., Stockholm, Sweden), which can carry out cell modeling and simulation coupling of multi-physical fields. It is a commonly used software for finite element analysis. COMSOL also has the following advantages [27]: (1) It can build two-dimensional, two-dimensional axisymmetric and three-dimensional models; (2) It can be meshed manually or automatically; (3) It can be associated with a variety of drawing software to achieve data interaction. According to the geometry and structure of the CMUT cell, an appropriate geometric model and meshing method can be selected to improve calculation efficiency. In addition, the results obtained from COMSOL simulation can be exported for further analysis and processing in MATLAB R2020a (The MathWorks, Inc., Natick, MA, USA).

In this paper, COMSOL is used to analyze the influence of the membrane material, membrane shape, membrane radius thickness, and cavity height on the performance of the Vacuum CMUT cell, which provides a basis for the structural design of the CMUT cell.

### 3.1. The Influence of Membrane Materials on the Deflection of Vacuum CMUT Cell

In this study, SI, SIC, SI3N4, and Diamond were selected as membrane materials for cell modeling. Table 1 shows the main performance parameters of the Vacuum CMUT cell structure.

**Figure 4:**
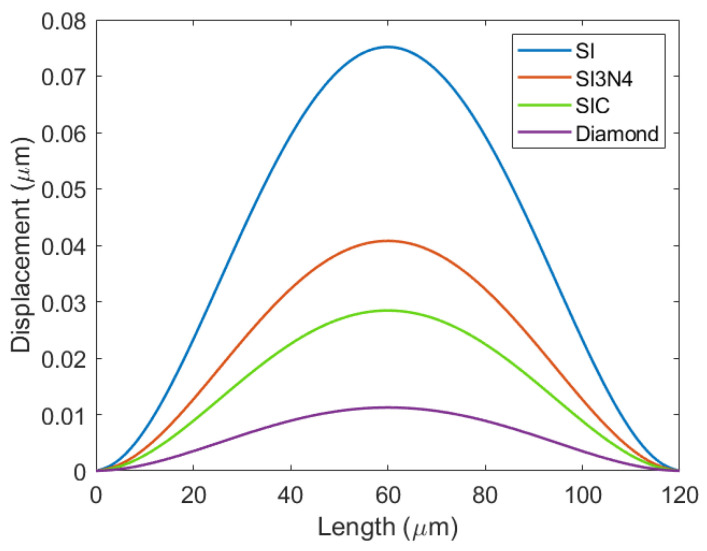
The membrane deflections of different materials.

After the CMUT cell model was established, the solid mechanics field was added for static simulation analysis, so as to obtain the vibration deflection of the Vacuum CMUT cell under different membrane materials, as shown in Figure 4.

The deflection of the Vacuum CMUT cell with the same membrane radius and thickness is different when different membrane materials are selected. It can be seen from Figure 4 that the SI membrane has the maximum deflection, while the Diamond membrane has the minimum deflection, which is determined by the bending stiffness of the material. SI has the minimum bending stiffness and is more likely to obtain a large deflection under the same conditions. Therefore, in order to increase the deflection and improve the efficiency of the CMUT, the SI membrane material should be used.

### 3.2. The Influence of Membrane Shape on Deflection of Vacuum CMUT Cell

In this study, Vacuum CMUT cells with circular, hexagonal, and square membranes were constructed respectively. Table 2 shows the main performance parameters of the designed CMUT cell membrane structure.

**Figure 5:**
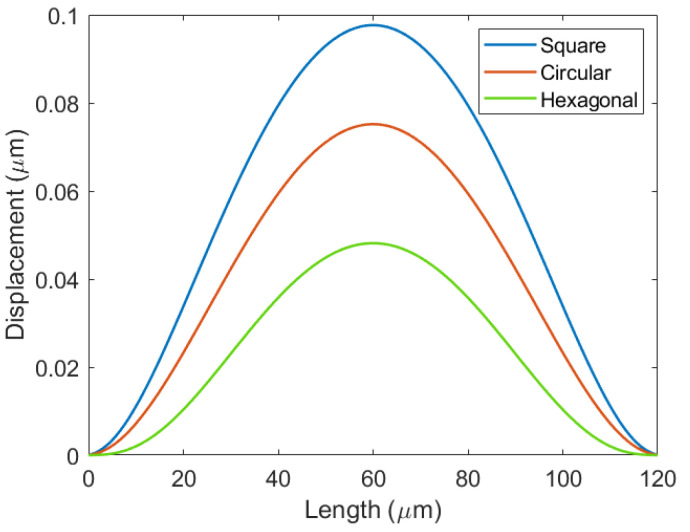
The membrane deflections of different shapes.

The deflection simulation results of the CMUT membrane with different shapes are shown in Figure 5.

When other parameters are the same, the shape of the membrane will affect the deflection of the Vacuum CMUT. Figure 5 shows that the deflection of the square membrane is the largest, the circular membrane is in the middle, and that of the hexagonal membrane is the smallest. This is because the square membrane has the largest fill factor and the hexagonal membrane has the smallest. In the design process of the Vacuum CMUT cell, the filling factor of the membrane should be increased as much as possible in order to increase the deflection. However, in practice, the effect of edge stress should also be considered. Under the same condition, the edge stress of the circular membrane CMUT affected by external force is the smallest. Taking the above factors into consideration, the circular membrane is adopted in this paper.

### 3.3. The Influence of Membrane Radius Thickness on the Performance of VACUUM CMUT

**Figure 6:**
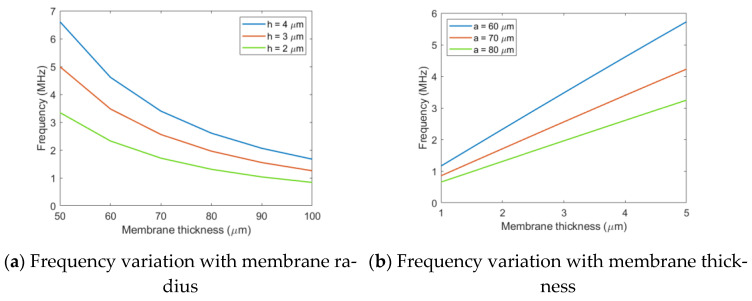
Frequency variation with membrane radius and membrane thickness.

On the basis of the above research, the circular SI membrane was selected as the research object. The influence of membrane radius thickness on the performance of the Vacuum CMUT was investigated, of which the influence on the frequency was the first studied. The frequency of the Vacuum CMUT membrane was obtained as a function of the membrane radius and thickness through simulation, as shown in Figure 6.

It can be seen from the above Figure 6 that when the membrane thickness is constant, its frequency decreases with the increase in radius. When the membrane radius is constant, its frequency gradually increases with the increase in thickness, which is consistent with the conclusion of Equation (10). In the design process of the Vacuum CMUT cell, different membrane radius thickness combinations can be selected to achieve a specific frequency. In this paper, the fixed frequency was 3 MHz, and three sets of radius thickness combinations of 60–2.6 μm, 70–3.5 μm, and 80–4.6 μm were selected to simulate and analyze the influences of different radius thickness combinations on Vacuum CMUT transmission sound pressure, reception sensitivity, and fractional bandwidth.

**Figure 7:**
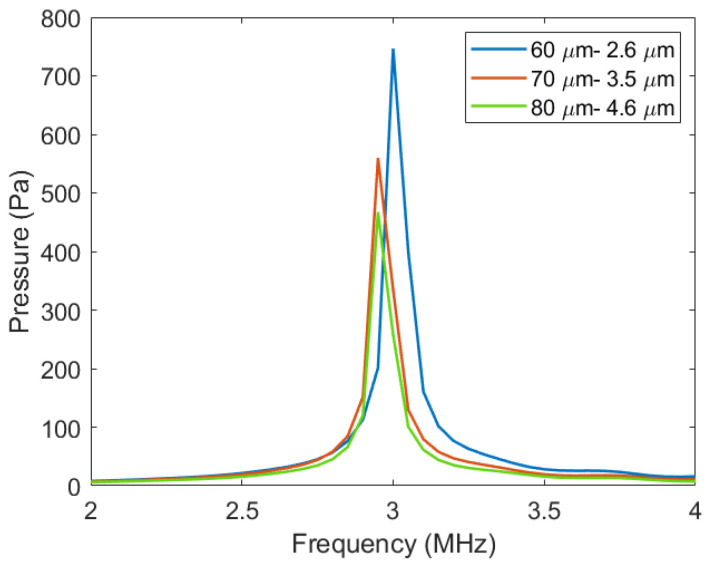
The sound pressure of different membrane radius thickness combinations.

In order to obtain the sound pressure characteristics of the Vacuum CMUT cell, a harmonic response simulation was carried out using COMSOL software. The pressure acoustic-frequency and electromechanical field were added to the established model. Through the coupling of the two physical fields, the resonant frequency and sound pressure of the cell can be calculated. Before the simulation, the collapse voltage of each cell was first calculated. In the harmonic response simulation, 80% of the collapse voltage was selected as the DC bias voltage to be added between the upper and lower electrodes, and the frequency and sound pressure of these cells were obtained through finite element calculation. Figure 7 shows the transmission sound pressure of different membrane radius thickness combinations.

Figure 7 shows that under the same other conditions, the transmission sound pressure of the Vacuum CMUT decreases with the increase in the membrane radius thickness.

In this paper, COMSOL simulation and MATLAB calculation were used to analyze the influence of different membrane radius thickness combinations on the sensitivity of the Vacuum CMUT. Firstly, the static capacitance of the CMUT cell was obtained through finite element simulation, and then, ultrasonic excitation was applied to the surface of the CMUT to obtain the dynamic capacitance in this state. Finally, the ratio of capacitance variation to the load was calculated by MATLAB, thus obtaining the sensitivity of CMUT. Table 3 shows the sensitivity of the Vacuum CMUT with different membrane radius thickness combinations.

It can be seen from the above table that the sensitivity of the Vacuum CMUT is related to the membrane radius thickness combination under the same other conditions. As the membrane radius thickness increases, the sensitivity of the CMUT gradually increases.

**Figure 8:**
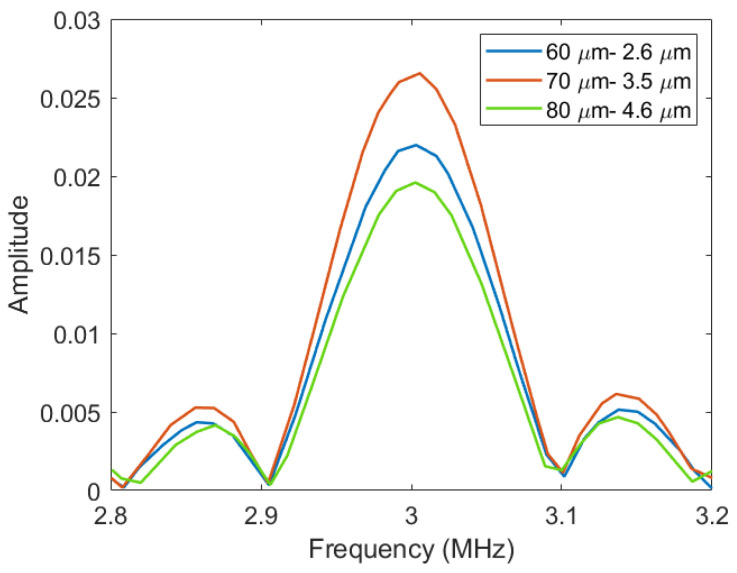
The fractional bandwidth of different membrane radius thickness combinations.

In order to obtain fractional bandwidth of the Vacuum CMUT, transient simulation was carried out. First, we established the CMUT cell model. A three-dimensional model was established to obtain accurate calculation results. Secondly, we set up the physical field. An electromechanical field with corresponding boundary conditions and initial conditions was added to the CMUT cell. Then, we selected the transient study. Finally, the variation of the CMUT membrane vibration with time was obtained. The data calculated by COMSOL were exported to MATLAB for Fourier transform processing, and the variation of CMUT membrane vibration with frequency was obtained. Then, the frequency corresponding to −3 dB amplitude was calculated, and finally, the fractional bandwidth of CMUT was obtained. Figure 8 shows the fractional bandwidth of different membrane radius thickness combinations.

It can be seen from Figure 8 above that under the same other conditions, the fractional bandwidth of the Vacuum CMUT gradually increases with the increase in membrane radius thickness.

Based on the above research, it can be seen that membrane radius thickness combinations will affect the transmission sound pressure, reception sensitivity, and fractional bandwidth of the Vacuum CMUT. With the increase in the membrane radius thickness, the sound pressure of the CMUT tends to decrease, while the sensitivity and bandwidth tend to increase. In the design process of the Vacuum CMUT cell, the appropriate membrane radius thickness should be selected according to the application. For example, when the CMUT is used as a transmitter, it is necessary to increase the transmission sound pressure as much as possible, so a smaller membrane should be selected. When the CMUT is used as a receiver, the receiving sensitivity needs to be increased as much as possible, so a larger membrane should be selected.

### 3.4. The influence of Cavity Height on the Performance of Vacuum CMUT

The cavity height is one of the important structural parameters of the Vacuum CMUT cell, which plays a vital role in the performance of the CMUT. In this paper, the membrane was fixed with a radius of 60 μm and a thickness of 2.6 μm, and the cavity height was selected to be 0.2 μm, 0.3 μm, and 0.4 μm, respectively. The influence of cavity height on the cell performance of the Vacuum CMUT was investigated.

**Figure 9:**
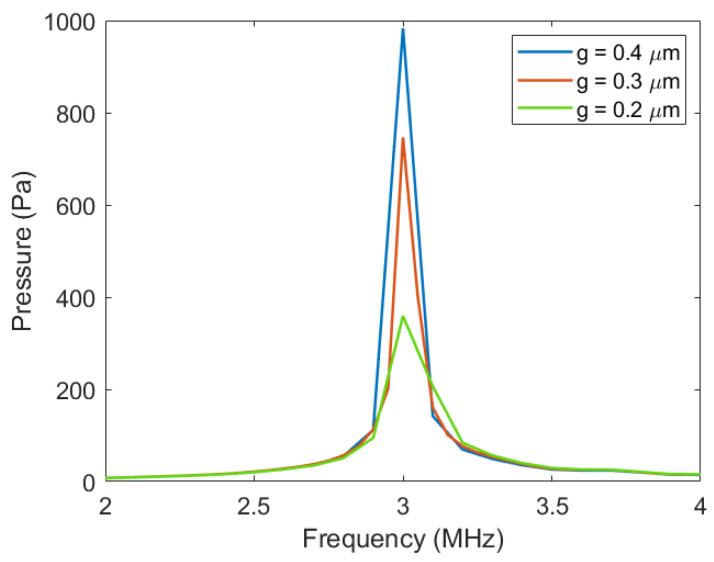
The sound pressure of different cavity heights.

Through COMSOL harmonic response simulation, under the coupling effect of pressure acoustics-frequency and electromechanical electric field, the transmission sound pressure of the Vacuum CMUT cell under different cavity heights was obtained, as shown in Figure 9.

It can be seen from Figure 9 that the transmission sound pressure of the Vacuum CMUT is greatly affected by the height of the cavity. In the case of all other parameters remaining the same, the transmission pressure of the CMUT increases with the increase in cavity height. This is because the higher the cavity height is, the larger the membrane deflection of the CMUT will be, and the higher the sound pressure generated by its vibration will be.

The static and dynamic capacitance of the CMUT cell were obtained by COMSOL finite element simulation, and then, the ratio of capacitance variation to load was calculated by MATLAB, and the sensitivity of the Vacuum CMUT with different cavity heights was obtained. Table 4 shows the sensitivity values of different Vacuum CMUTs.

The data in the above table show that the sensitivity of the Vacuum CMUT decreases with the increase in cavity height. This is because when all the other conditions are the same, the higher the cavity height, the smaller the dynamic capacitance variation caused by external sound pressure excitation, and thus the smaller the sensitivity of CMUT. This is consistent with the conclusion shown in Equation (20).

**Figure 10:**
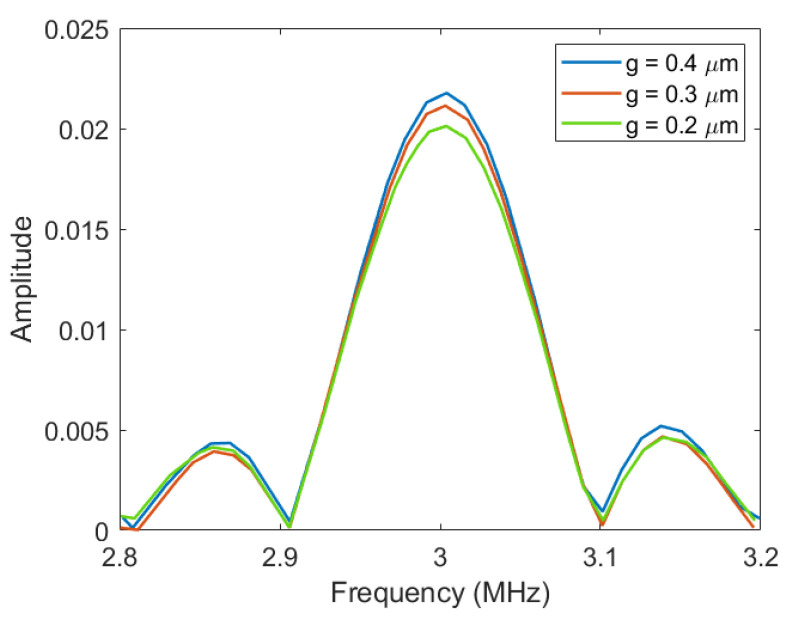
The fractional bandwidth of different cavity heights.

Through the transient simulation of COMSOL and the Fourier calculation of MATLAB, the fractional bandwidth of the Vacuum CMUT under different cavity heights was obtained. The result is shown in Figure 10.

It can be seen from Figure 10 that the fractional bandwidth of the Vacuum CMUT does not change significantly with the increase in the cavity height. This indicates that the fractional bandwidth of the CMUT is less affected by the cavity height. When a specific fractional bandwidth needs to be realized, other structural parameters should be considered.

Through the above analysis, it can be found that the cavity height of the Vacuum CMUT will mainly affect its transmission sound pressure and reception sensitivity. Under the same other conditions, increasing the cavity height of the Vacuum CMUT will increase its transmission sound pressure and reduce its reception sensitivity. It can be seen that the transmission sound pressure and the reception sensitivity are a pair of mutually restrictive parameters. Increasing one of them will reduce the other one. In the process of designing the cell structure of the Vacuum CMUT, these two parameters should be considered comprehensively to select the appropriate cavity height.

## 4. Conclusions

In this paper, the structure of the Vacuum CMUT is presented, and its performance-influencing factors are analyzed. The paper first derives the equations of each performance parameter of the Vacuum CMUT, analyzes the influencing factors of these performance parameters from the theoretical aspect, and proposes the design principles of the Vacuum CMUT cell structure. Then, COMSOL finite element simulation software and MATLAB calculation software are used to further analyze the influence of the structural parameters on its performance. The results show that: (1) In order to obtain the Vacuum CMUT with large deflection, the membrane material with small bending stiffness should be selected, and the filling factor of the membrane should be increased. (2) In order to obtain the Vacuum CMUT with high transmission pressure, a smaller radius thickness and a larger cavity height should be selected. (3) In order to obtain the Vacuum CMUT with high reception sensitivity, a larger membrane radius thickness and a smaller cavity height should be selected. (4) In order to obtain the Vacuum CMUT with high fractional bandwidth, a larger membrane radius thickness should be selected. The analysis results in this paper provide a basis for the selection of parameters of Vacuum CMUT cell structure and the optimization of its performance.

## Figures and Tables

**Table 1:** Performance parameters of different membrane materials.

Membrane Parameters	SI	SIC	SI3N4	Diamond
Radius (μm)	60	60	60	60
Thickness (μm)	2.6	2.6	2.6	2.6
Young’s modulus (Gpa)	169	476	320	1200
Poisson’s ratio	0.299	0.19	0.26	0.2
Density (kg/m^3^)	2332	3210	3270	3520

**Table 2:** Performance parameters of different membrane shapes.

Membrane Parameters	Circular	Square	Hexagonal
Radius (μm)	60	120	60
Thickness (μm)	2.6	2.6	2.6
Young’s modulus (Gpa)	169	169	169
Poisson’s ratio	0.299	0.299	0.299
Density (kg/m^3^)	2332	2332	2332

**Table 3:** The sensitivity of different membrane radius thickness combinations.

Radius Thickness (μm)	Sensitivity (F/kPa)
60–2.6	5 × 10^−15^
70–3.5	1.7 × 10^−14^
80–4.6	1.9 × 10^−14^

**Table 4:** The sensitivity of different cavity heights.

Cavity Height (μm)	Sensitivity (F/kPa)
0.2	5.51 × 10^−15^
0.3	5 × 10^−15^
0.4	4.66 × 10^−15^

## Data Availability

Not applicable.
